# Case Report: Emphysematous Cystitis and Pyelonephritis Induced by Uterine Prolapse in a Subject With Untreated Diabetes Mellitus

**DOI:** 10.3389/fmed.2021.658682

**Published:** 2021-04-09

**Authors:** Hideyuki Iwamoto, Takatoshi Anno, Haruka Takenouchi, Kaio Takahashi, Megumi Horiya, Yukiko Kimura, Fumiko Kawasaki, Kohei Kaku, Koichi Tomoda, Shinya Uehara, Hideaki Kaneto

**Affiliations:** ^1^Department of General Internal Medicine, Kawasaki Medical School, Kawasaki, Japan; ^2^Department of Urology, Kawasaki Medical School, Kawasaki, Japan; ^3^Department of Diabetes, Endocrinology and Metabolism, Kawasaki Medical School, Kawasaki, Japan

**Keywords:** emphysematous cystitis, emphysematous pyelonephritis, uterine prolapse, diabetes mellitus, obstructive uropathy

## Abstract

Type 2 diabetes mellitus (T2DM) is often accompanied by a lot of complications due to chronic hyperglycemia and inflammation. Emphysematous cystitis and pyelonephritis are rare types of urinary tract infections and are often complicated with DM. Herein, we report a case of emphysematous cystitis and pyelonephritis complicated with untreated DM. In addition, this case was very rare and interesting in that her emphysematous cystitis and pyelonephritis were induced by severe uterine prolapse, obstructive uropathy and urination disorders. Both uterine prolapse and DM should be appropriately treated because both can lead to the development of emphysematous cystitis and pyelonephritis.

## Introduction

Type 2 diabetes mellitus (T2DM) is often accompanied by a lot of complications due to chronic hyperglycemia and inflammation. Emphysematous cystitis and pyelonephritis are rare types of urinary tract infections and are often complicated with DM (1, 2). In addition, the most causative bacteria are *Escherichia coli* which can cause septic shock and/or disseminated intravascular coagulation (DIC) and sometimes lead to death. Also, urination disorders such as neurogenic bladder complicated with DM can lead to the development of emphysematous cystitis and pyelonephritis. On the other hand, uterine prolapse can bring out various urination disorders. Especially, severe uterine prolapse leads to the development of obstructive uropathy and end-stage renal disease (3, 4).

## Case Description

**Figure 1:**
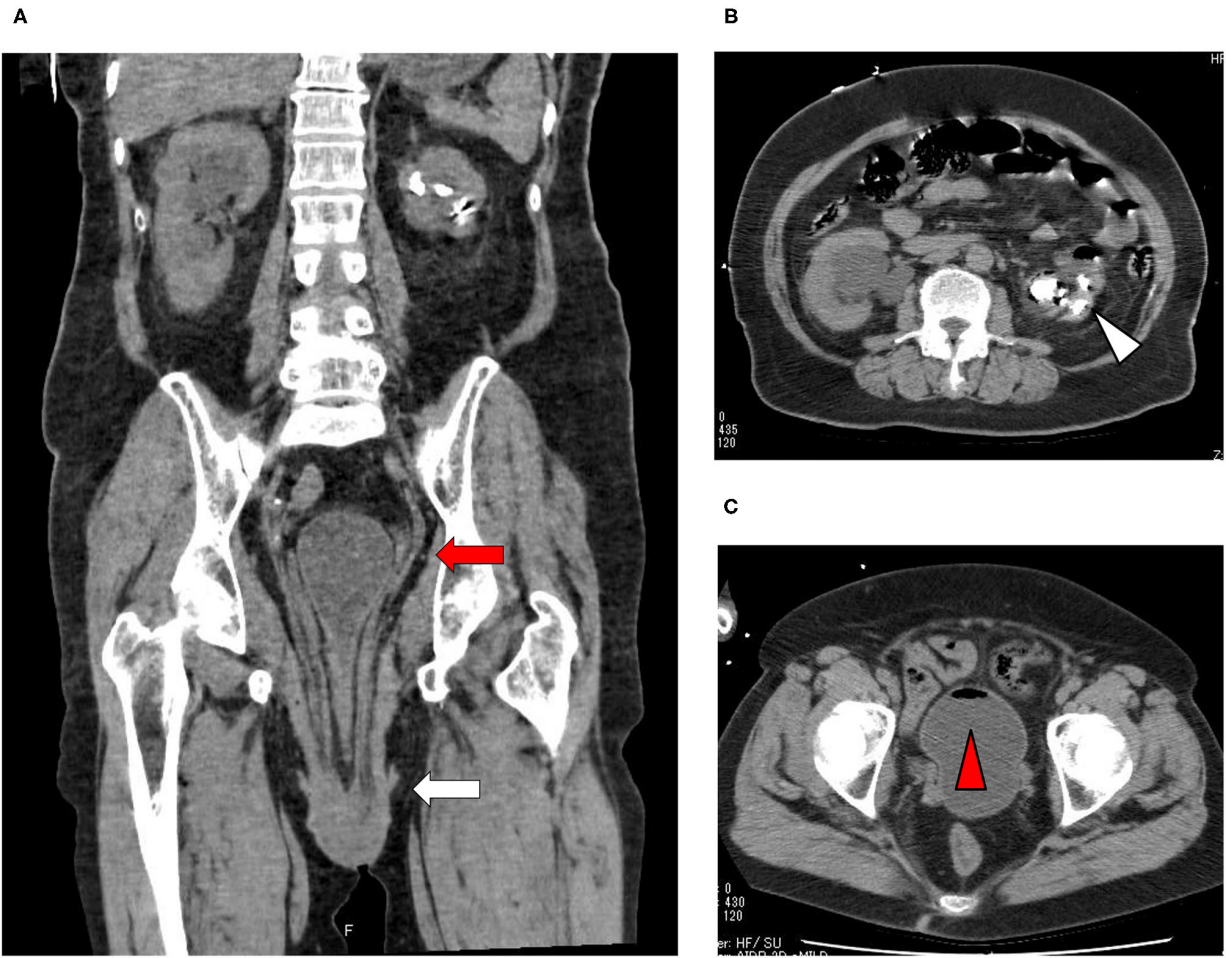
**(A)** Abdominal CT revealed renal calculus and hydronephrosis, and pelvic CT revealed lower shift of bladder (red arrow) and severe uterine prolapse (white arrow). **(B,C)** Abdominal CT showed emphysematous pyelonephritis (white triangle), and pelvic CT showed emphysematous cystitis (red triangle).

**Figure 2:**
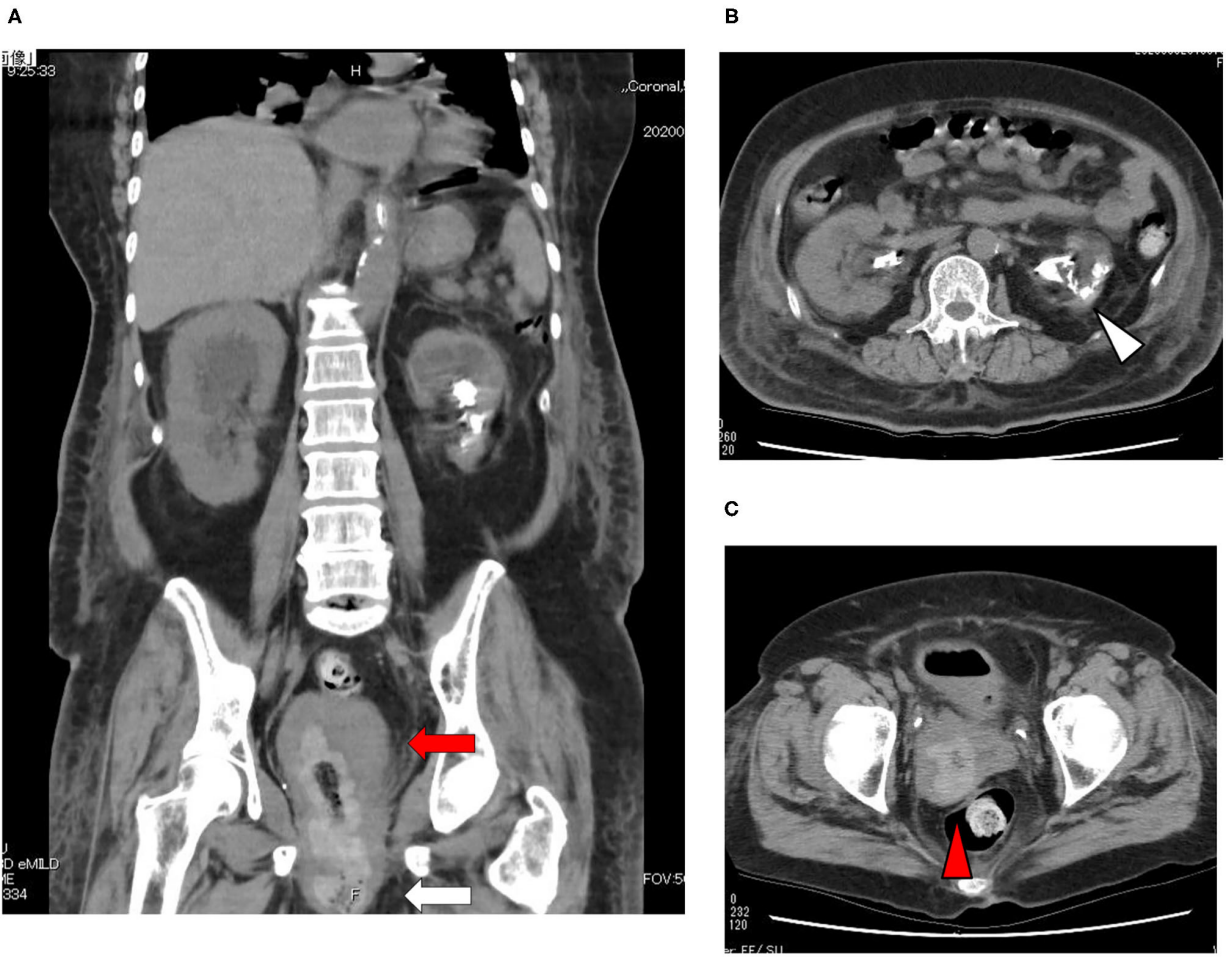
**(A–C)** Abdominal and pelvic CT taken 10 days after admission. Abdominal and pelvic CT revealed that hydronephrosis was improved, although renal calculus was observed. In addition, pelvic CT revealed that lower shift of bladder (red arrow) and severe uterine prolapse (white arrow) were improved.

**Figure 3:**
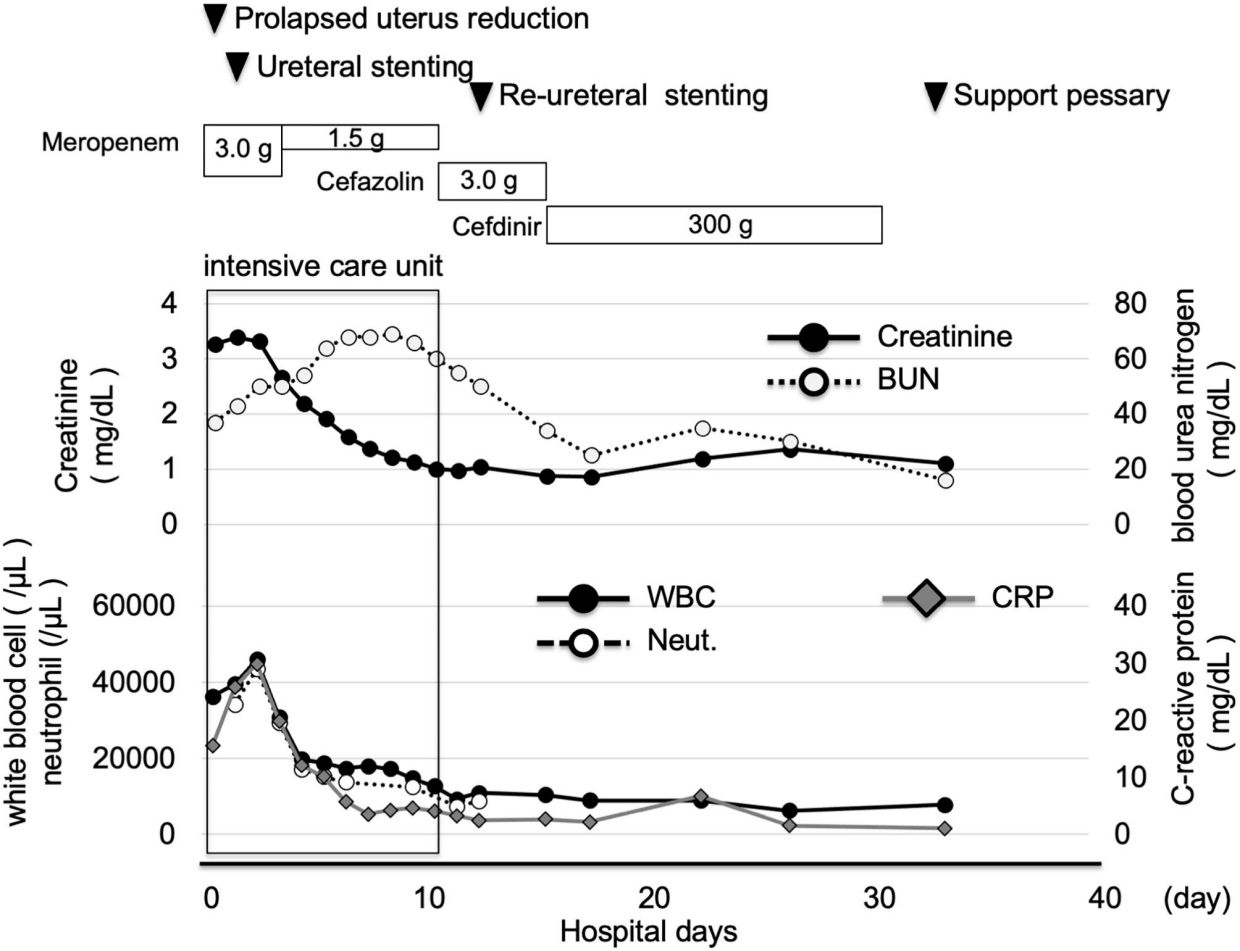
Time course of clinical parameters in this subject. After prolapsed uterus reduction, ureteral stenting and starting antibiotics, her inflammation markers were markedly improved. She was transferred from intensive care unit to general ward at day 10. After then, her renal function and inflammatory markers were gradually normalized and she was finally discharged about 1 month after admission.

A 73-year-old Japanese woman was brought to an emergency room due to difficulty in urinating. At the age of 70, she was diagnosed with complete eversion of uterine prolapse and type 2 diabetes mellitus (T2DM), but she hesitated to get any medical treatment. Her vital signs were as follows: temperature, 36.8°C; blood pressure, 96/50 mmHg; heart rate, 30 beats/min; oxygen saturation, 84 % (room air). Table 1 shows laboratory data in an emergency room. Diabetes-associated data were as follows: plasma glucose, 241 mg/dl; hemoglobin A1c (HbA1c), 9.3%. Liver and renal function was markedly elevated as follows: asparate aminotransferase (AST), 567 U/L; alanine transaminase (ALT), 196 U/L; alkaline phosphatase (ALP) 214 U/L; γ-glutamyl transpeptidase (γ-GTP), 33 U/L; lactate dehydrogenase (LDH), 1670 U/L; creatinine (CRE), 3.27 mg/dl; blood urea nitrogen (BUN), 37 mg/dl. In addition, her inflammation markers were markedly elevated probably due to the development of DIC: white blood cell, 36,260 /μL (neutrophil, 93.0%); C-reactive protein, 15.54 mg/dl; procalcitonin, 278.70 Ng/mL; platelets, 9.2 × 10^4^/μL; prothrombin percentage activity, 65.8%; fibrinogen, 288 mg/dl; D-dimer, 69.60 μg/mL; antithrombin III activity, 62.5%. As shown in Figure 1A, her abdominal and pelvic computed tomography (CT) on admission revealed renal calculus, hydronephrosis, lower shift of bladder, urinary tract and severe uterine prolapse. In addition, as shown in Figures 1B,C, she suffered from emphysematous cystitis and pyelonephritis both of which contained some gas inside the organ. Same pathogenic bacteria (*Escherichia coli*) were detected in blood and urine. Since we thought that uterus prolapse was possibly involved in the aggravation of the conditions in this subject, we performed prolapsed uterus reduction and ureteral stenting. In addition, we also started antibiotics therapy for emphysematous cystitis and pyelonephritis (3 g/day of meropenem) and treated DIC. In abdominal and pelvic CT taken 10 days after admission, hydronephrosis was improved, although renal calculus was observed. In addition, pelvic CT revealed that lower shift of bladder (red arrow) and severe uterine prolapse (white arrow) were improved (Figures 2A–C). Finally, we successfully treated emphysematous cystitis and pyelonephritis, and she was transferred from intensive care unit to general ward at day 10. After then, we stopped meropenum and started 3.0 g/day of cefazolin, and after several days later we changed cefazolin to 300 g/day of cefdinir (Figure 3). Her renal function and inflammatory markers were gradually normalized and she was finally discharged about 1 month after admission.

**Table 1:** Laboratory data in an emergency room in this subject.

**Variable**	**Result**	**Reference range**	**Variable**	**Result**	**Reference range**
**Peripheral blood**			**Diabetes marker**		
White blood cells (/μL)	36,260	3,300–8,600	Plasma glucose (mg/dL)	241	
Neutrophil (%)	93.0	28.0–78.0	Hemoglobin A1c (%)	9.3	4.9–6.0
Red blood cells (× 10^4^/μL)	446	435–555	**Infectious marker**		
Hemoglobin (g/dl)	13.5	13.7–16.8	CRP (mg/dl)	15.54	<0.14
Hematocrit (%)	40.7	35.1–44.4	Procalcitonin (ng/mL)	278.70	0.00–0.05
Platelets (× 10^4^/μL)	9.2	15.8–34.8	**Blood Gas Analysis**		
**Blood biochemistry**			pH	7.343	7.360–7.460
Total protein (g/dl)	6.5	6.6–8.1	PCO_2_ (mmHg)	22.5	34.0–46.0
Albumin (g/dl)	3.1	4.1–5.1	PO_2_ (mmHg)	56.9	80.0–90.0
Globulin (g/dl)	3.4	2.2–3.4	${\text{HCO}}_{3}^{-}$ (mEq/L)	11.9	24.0–32.0
Total bilirubin (mg/dl)	0.5	0.4–1.5	BE (mEq/L)	−11.8	−2.5–2.5
AST (U/L)	567	13–30	SO_2_ (%)	87.7	95.0–98.0
ALT (U/L)	196	10–42	Lactate (mEq/L)	7.50	0.63–2.44
LDH (U/L)	1670	124–222	**Coagulation fibrinolytic system-related antibodies**		
ALP (U/L)	214	106–322	PT-sec (sec)	14.7	9.3–212.5
γ-GTP (U/L)	33	13–64	PT-INR	1.23	0.85–1.13
BUN (mg/dl)	37	8–20	PT-activity (%)	65.8	80.7–125.2
Creatinine (mg/dl)	3.24	0.65–1.07	APTT (sec)	36.7	26.9–38.1
Cholinesterase (U/L)	259	240–486	Fibrinogen (mg/dl)	288	160–380
Uric acid (mg/dl)	11.0	2.6–5.5	D-dimer (μg/mL)	69.60	<1.0
Creatine Kinase (U/L)	40506	41–153	Antithrombin III activity (%)	62.5	82.0–132.0
Amylase (μg/dl)	3109	42–118			
Total cholesterol (mg/dl)	149	142–248			
Sodium (mmol/L)	138	138–145			
Potassium (mmol/L)	3.4	3.6–4.8			
Chloride (mmol/L)	97	101–108			

*AST, aspartate aminotransferase; ALT, alanine aminotransferase; LDH, lactate dehydrogenase; ALP, alkaline phosphatase; γ-GTP, γ-glutamyl transpeptidase; BUN, blood urea nitrogen; CRP, C-reactive protein; BE, Base Excess; PT, prothrombin; PT-INR, PT-international normalized ratio; APTT, activated partial thromboplastin time*.

## Discussion

Herein we report a case of emphysematous cystitis and pyelonephritis which were complicated with untreated DM. In addition, this case is very rare and interesting in that her emphysematous cystitis and pyelonephritis were induced by severe uterine prolapse, obstructive uropathy and urination disorders. Main causes of emphysematous cystitis and pyelonephritis are DM, catheter use, chronic urinary tract infections, being elderly female and neurogenic bladder (5). However, precise pathogenesis of emphysematous cystitis and pyelonephritis remains unclear. This patient had poorly controlled T2DM, but she hesitated to get any medical treatment. The development of emphysematous pyelonephritis is often associated with uncontrolled DM (6). Although her glycemic control was also poor, we think her emphysematous cystitis and pyelonephritis were, at least in part, associated with obstructive uropathy and urination disorders which were caused by severe uterine prolapse. Indeed, her emphysematous cystitis and pyelonephritis were improved after prolapsed uterus reduction and ureteral stenting. Concerning DM, she was temporarily treated with insulin preparation, and finally she was treated with oral anti-diabetic drugs (5 mg/day of linagliptin and 500 mg/day of metformin).

Severe uterine prolapse is caused by obstructive uropathy and urination disorders. In addition, severe uterine prolapse can lead to the development of obstructive uropathy and end-stage renal disease (3, 4). Our patient suffered from severe uterine prolapse at least for 3 years, which finally became complete eversion. Her obstructive uropathy and urination disorders were severe because her abdominal CT revealed renal calculus and hydronephrosis, which was probably due to long-time obstructive uropathy and urination disorders complicated with severe uterine prolapse. Emphysematous cystitis and pyelonephritis are caused by obstructive uropathy and urination disorders. Therefore, we think her emphysematous cystitis and pyelonephritis were, at least in part, associated with severe uterine prolapse.

We think that there are new medical knowledge and educational value in this case. First, to the best of our knowledge, this is the first report showing a case with emphysematous cystitis and pyelonephritis which were induced by uterine prolapse. Second, it is likely that in this case emphysematous cystitis and pyelonephritis were caused by poorly controlled DM and obstructive uropathy. Therefore, it is possible that obstructive uropathy and urination disorders including ureteral stones, prostatic hypertrophy, uterine prolapse lead to the development of emphysematous cystitis and pyelonephritis, especially in subjects with poorly controlled DM.

## Conclusion

Taken together, we should bear in mind that emphysematous cystitis and pyelonephritis could be caused by obstructive uropathy and urination disorders as well as poorly controlled DM. In addition, this case is very interesting in that we successfully treated emphysematous cystitis and pyelonephritis not only with antibiotics and ureteral stenting but also with prolapsed uterus reduction. Both uterine prolapse and DM should be appropriately treated because both can lead to the development of emphysematous cystitis and pyelonephritis.

## Data Availability Statement

The original contributions generated for the study are included in the article/supplementary material, further inquiries can be directed to the corresponding authors.

## Ethics Statement

Written informed consent was obtained from the individual's for the publication of any potentially identifiable images or data included in this article.

## Author Contributions

HI and TA researched data and wrote the manuscript. HT, KTa, MH, YK, FK, SU, and KTo researched data and contributed to the discussion. KK and HK reviewed the manuscript. All authors contributed to the article and approved the submitted version.

## Conflict of Interest

The authors declare that the research was conducted in the absence of any commercial or financial relationships that could be construed as a potential conflict of interest.
